# Myasthenia Gravis and COVID-19: Clinical Characteristics and Outcomes

**DOI:** 10.3389/fneur.2020.01053

**Published:** 2020-09-11

**Authors:** Antonio E. Camelo-Filho, André M. S. Silva, Eduardo P. Estephan, Antônio A. Zambon, Rodrigo H. Mendonça, Paulo V. S. Souza, Wladimir B. V. R. Pinto, Acary S. B. Oliveira, Iron Dangoni-Filho, Ana F. P. Pouza, Berenice C. O. Valerio, Edmar Zanoteli

**Affiliations:** ^1^Department of Neurology, Faculdade de Medicina da Universidade de São Paulo, São Paulo, Brazil; ^2^Department of Neurology, Faculdade de Medicina Santa Marcelina, São Paulo, Brazil; ^3^Department of Neurology and Neurosurgery, Universidade Federal de São Paulo, São Paulo, Brazil; ^4^Department of Neurology, Instituto de Assistência Médica ao Servidor Público Estadual, São Paulo, Brazil; ^5^Department of Internal Medicine, Faculdade de Ciências Médicas da Santa Casa de São Paulo, São Paulo, Brazil

**Keywords:** Myasthenia Gravis, COVID-19, SARS-CoV-2, neuromuscular disorders, immunosuppression

## Abstract

Myasthenia gravis (MG), an autoimmune neuromuscular disorder, may be a risk factor for severe COVID-19. We conducted an observational retrospective study with 15 consecutive adult MG patients admitted with COVID-19 at four hospitals in São Paulo, Brazil. Most patients with MG hospitalized for COVID-19 had severe courses of the disease: 87% were admitted in the intensive care unit, 73% needed mechanical ventilation, and 30% died. Immunoglobulin use and the plasma exchange procedure were safe. Immunosuppressive therapy seems to be associated with better outcomes, as it might play a protective role.

## Introduction

Since the first outbreak description of coronavirus disease 2019 (COVID-19) (1), there has been growing evidence of potential neurological complications of severe acute respiratory syndrome coronavirus 2 (SARS-CoV-2) (2). On the other hand, the current COVID-19 pandemic may impact specific neurological populations, such as neuromuscular and autoimmune disease patients, raising concerns regarding best practices in these groups.

Myasthenia gravis (MG), an autoimmune neuromuscular disorder, may be a risk factor for severe COVID-19 due to multiple issues, such as immunosuppressive therapy, baseline respiratory weakness, and exacerbation from a viral infection and drug exposure (3). However, the available information is limited. Only one study (4) describing a series of five patients and two case reports (5), including a myasthenic crisis and a patient with a mild COVID-19 course (6), were reported. Here, we describe characteristics and outcomes of 15 hospitalized patients with MG and COVID-19.

## Method

We conducted an observational retrospective cross-sectional study including all consecutive adult patients diagnosed with MG (based on antibodies or on electrophysiology) and admitted with COVID-19 at four hospitals in São Paulo, Brazil, from March 15, 2020, to May 31, 2020. All patients had COVID-19 diagnoses based on respiratory symptoms and on positive nasopharyngeal swab polymerase-chain-reaction (RT-PCR) testing for SARS-CoV-2. Informed consent was waived because of the retrospective observational nature of the study and the analysis used anonymous clinical data.

All patients underwent detailed clinical examinations, and neuromuscular specialists in each hospital collected the medical chart reviews. The MGFA (Myasthenia Gravis Foundation of America) scores (7) were defined based on the clinical descriptions one month prior to hospitalizations. MG exacerbation was defined as a MGFA score worsening from the baseline or a respiratory insufficiency needing mechanical ventilation (MV). We considered a use of at least 40 mg prednisone per day or an association of prednisone plus a second immunosuppressant drug as a high level of immunosuppressive treatment.

## Results

Fifteen patients with MG and COVID-19 were identified, including 10 patients with the anti-acetylcholine receptor (AChR) antibody, one patient with the anti-muscle-specific tyrosine kinase (MuSK) antibody, and four patients without serological definition. Table 1 summarizes the clinical characteristics, treatments, and outcomes of the patients.

**Table 1:** Clinical characteristics, treatments and outcomes of the 15 patients with myasthenia gravis and COVID-19.

**#**	Age group*	**MGFA pre-adm**	**Years of MG**	**Antibody**	**Baseline immunosuppressive treatment**	**Thymectomy**	**Commorbidities**	**MG symptoms exacerbation**	**Respiratory support**	**Chest CT > 50% involvement**	**cNMB**	**COVID19 treatments**	**MG treatments**	**days of ICU / total hospital stay**	**MGFA discharge**
1	≥60	I	15	AchR+	Pred 20 qd	No	None	Exacerbation leading to MV	MV	**	Yes	CTX, AZM, OTV, AMK, TEC	Pred continued	12/13	Deceased
2	≥60	I	3	AchR+	Pred 30 qd	No	DM, HTN	Exacerbation leading to MV	MV	Yes	Yes	CTX, AZM, TZP	Pred continued	8/9	Deceased
3	20–39	IIA	13	AchR+	Pred 60 qd + AZA 250 qd + IVIg 1g/kg monthly	Yes	None	Exarcerbation without MV	NC	No	No	CTX	Pred and AZA continued, IVIg 2g/kg added	4/8	IIA
4	40–59	IIA	10	AchR+	Pred 30 qd + MTX 15 mg weekly	Yes	DM	No	NC	No	No	CTX	Pred increased to 40 mg/day, MTX continued	16/18	Remains hospitalized (IIA)
5	20–39	IIA	4	N/A	Pred 5 qd + MTX 20 mg weekly	No	HTN, SLE	Exacerbation leading to MV	MV	No	Yes	CTX, OTV, MEM, CST, LZD	Pred continued, MTX withheld	25/29	Remains hospitalized (V)
6	40–59	IIA	14	N/A	No therapy	Yes	None	Exacerbation leading to MV	MV	Yes	Yes	CLR, CTX, AZM, OTV	None	7/7	Deceased
7	40–59	IIA	9	AchR+	Pred 40 qd + CCP 150 mg qd	Yes	HTN, DM, HCV	Exacerbation leading to MV	MV	No	No	CTX, AZM, TZP, MEM	Pred increased, 5 PLEX sessions added, CCP withheld	25/42	IIA
8	40–59	IIB	22	AchR+	Pred 10 qd + AZA 200 qd	Yes	GAD	Exacerbation leading to MV	MV	No	No	CTX, AZM, TZP	Pred increased, 4 PLEX sessions added, AZA withheld	20/24	IIA
9	20–39	I	5	AchR+	Pred 5 qd + AZA 200 qd	No	DM, Epilepsy	Exacerbation leading to MV	MV	Yes	No	CTX, AZM	Pred continued, 5 PLEX sessions added, AZA withheld	12/20	IIA
10	20–39	IIB	2	Musk+	Pred 60 qd + RTX 375mg/m2	No	None	Exacerbation leading to MV	MV	No	No	CTX, AZM	Pred continued, 5 PLEX sessions added	16/28	IIB
11	20–39	I	11	AchR+	Pred 5 qd + AZA 150 qd	No	None	No	NC	No	No	AZM	Pred increased to 20mg/day, AZA continued	0/3	I
12	≥60	I	5	N/A	Pred 25 qd	No	HTN, asthma	Exacerbation leading to MV	MV	Yes	Yes	CTX, CLR, MEM, VAN	Pred increased	15/16	Deceased
13	20–39	IIB	16	N/A	Pred 30 qd + AZA 250 qd	Yes	None	Exarcerbation without MV	NC	No	No	CTX, AZM	Pred increased to 60 mg/day and AZA withheld	0/8	IIB
14	≥60	III	1	AchR+	Pred 40 qd + IVIg 2g/kg IBW monthly	No	HTN	Exacerbation leading to MV	MV	Yes	No	CTX, AZM, MEM, LZD, AMK, PMB	Pred increased	32/42	III
15	20–39	IIA	4	AchR+	Pred 30 qd	No	None	Exacerbation leading to MV	MV	No	Yes	CTX	Pred increased	17/22	I

AMK, Amikacin; AZA, Azathyoprine; AZM, Azithromycin; CCP, Cyclosporin; CLR, Clarithromycin; CST, Colistin; CTX, Ceftriaxone; cNMB, continuous neuromuscular blocking; GAD, generalized anxiety disorder; IVIg, Intravenous immunoglobulin; LZD, Linezolid; MEM, Meropenem; MGFA pre-adm, Myasthenia Gravis Foundation of America score at the month before admission; MTX, Methotrexate; MV, mechanical ventilation; OTV, Oseltamivir; PLEX, Plasma exchange; PMB, Polymyxin B; Pred, Prednisone; RTX, Rituximab; TEC, Teicoplanin; TZP, Piperacillin-tazobactam; VAN, Vancomycin. ^*^Written consent for gender and age information could not be obtained due to pandemic. Age presented in the following groups: 20–39 years, 40–59 years, and 60 years and over. ^**^Not performed due to the critical medical condition.

Nine (60%) female patients, with a mean age of 34.5 years, and six (40%) male patients, with a mean age of 61.3 years, were included. Ten patients presented generalized MG manifestations (MGFA score ≥ II) in the last month before admission, and the other five had only ocular symptoms (MGFA I). The median disease duration was 9 years. Fourteen (93.3%) patients were using prednisone, nine (60%) patients were taking a second oral immunosuppressant, one patient was receiving rituximab, and another patient was using monthly intravenous immunoglobulin (IVIG). Six (40%) patients were previously thymectomized.

Dyspnea (93.3%), fever (86.7%), cough (66.7%), and myalgia (46.7%) were the most common presenting symptoms. Thirteen (86.7%) patients needed hospitalization in an intensive care unit (ICU), with a median stay of 16 days. Most patients had MG exacerbation or mechanical ventilation needs. Eleven (73.3%) patients required intubation, and the remaining four (26.7%) needed an oxygen nasal cannula. Five patients requiring MV presented mild to moderate pneumonia (<50% pulmonary involvement). Two patients had worsening of MG symptoms without the need for mechanical ventilation support. The median hospitalization stay was 18 days, but two patients remained hospitalized at the time of the manuscript writing. Four patients died.

All patients received antibiotics (11 used a macrolide). Five (33.3%) patients were treated with specific therapies for MG exacerbation: one patient received IVIG, and four patients underwent plasma exchange therapy (PLEX). No complications regarding these therapies were reported. Six (40%) patients underwent continuous neuromuscular blockades (NMBs) for MV, and four of these patients died (#1, 2, 6, and 12) and one was still on mechanical ventilation (#5) for more than 14 days, at the time of the manuscript writing.

Of the four patients who died, all were male, more than 50 years old, did not use high levels of immunosuppressive treatment, and did not receive IVIG nor PLEX during hospitalization. Three of them had MGFA I before admission, and two had no other comorbidities. Otherwise, four (26.7%) patients had better outcomes without the need for MV. They were all young females using prednisone plus a second immunosuppressant drug. Additionally, a thymectomy did not necessarily determine a poor outcome because the disease course was similar between thymectomized and non-thymectomized patients.

## Discussion

To our knowledge, this is the largest series of MG and COVID-19 patients. Most patients hospitalized for COVID-19 with previous MG had a severe course of the disease (87% were admitted in ICU, and 73% needed mechanical ventilation). Previous use of prednisone plus immunosuppressive therapies did not seem to determine an additional unfavorable outcome. Lethality could not be completely determined in our study because two patients remained hospitalized at the time of the manuscript writing, but it was at least 30%.

Neurological complications of COVID-19 have been described in several studies and include encephalopathy, myalgia, headache, cerebrovascular disease, immune-mediated neuropathy, and rhabdomyolysis (2, 3, 8). However, the risk of worse outcomes for several groups of patients with autoimmune neurological diseases is still in debate, and few original studies addressed this issue. As long as the knowledge regarding the SARS-CoV-2 infection continues to evolve, several case reports and case series will try to answer whether some neurological or autoimmune conditions determine an unfavorable course of COVID-19 (3–7).

Moreover, using immunosuppressant drugs during the COVID-19 pandemic remains a challenge (9). Immunosuppressed patients could be at a higher risk for a more severe COVID-19 course. However, growing evidence shows that immunosuppression might play a protective role, reducing the immune response that leads to an inflammatory cytokine storm and to clinical deterioration (10, 11). There is a recent report of a multiple sclerosis patient (12) who had a favorable outcome after a COVID-19 infection while using a B cell-depleting drug: ocrelizumab. Our data support this hypothesis because the previous use of prednisone plus immunosuppressive therapies did not seem to cause an additional unfavorable outcome. Of the four patients that died, none used a high level of immunosuppressive treatment. Interestingly, all patients that did not require mechanical ventilation were using prednisone plus a second immunosuppressant drug. Previous small reports of MG and COVID-19 also demonstrated a favorable course in the patients using prednisone plus a second immunosuppressive drug at baseline (4, 6).

Regarding treatment for MG exacerbation, four patients underwent PLEX therapy and one patient received IVIG, and all of them had favorable outcomes. None of the patients that underwent these therapies died or had complications, and all patients, but one, were discharged without worse MGFAs compared to their admission scores. In other two reports, IVIG therapy was also administered for three MG patients with COVID-19, that evolved with favorable outcomes (4, 5). Furthermore, patients with MG frequently worry about exacerbation after exposure to drugs, such as antibiotics and NMB agents. In our series, the continuous use of NMBs was noted in most ventilated patients, and all of them died or remained hospitalized. This finding may confirm concerns about using these agents in MG patients.

Studies with COVID-19 patients requiring hospitalization revealed ICU admission rates from 14 to 26% and intra-hospital mortality rates from 21 to 28% (13, 14). Data from the Brazilian Ministry of Health shows an ICU admission rate of 32.9% and a mechanical ventilation rate of 18.6% throughout the country (15). Our cohort presents a rate of 86.7% of ICU admission and a lethality rate of 30%, although it could not be completely determined because two patients remained hospitalized. The more severe course in our cohort may relate to complex respiratory failure triggered by viral replication and MG exacerbation. Differentiating these possible causes is difficult, so neurological consultation and possibly early immunotherapy (IVIG and PLEX) for MG patients with severe COVID-19 infections are needed.

Our study has some limitations. First, this observational study included only hospitalized patients. We did not address the impact of MG and COVID-19 in outpatient settings. Second, the unfavorable course may be associated with other variables, such as age, comorbidities or pulmonary impairment. The deceased patients from our cohort had similar risk factors than non-MG fatal patients affected by COVID-19 (they were male, older than 60 years or had comorbidities). Thus, we cannot make assumptions that MG is an independent risk factor for death. The small number of patients and the absence of a control group limited statistical analysis for risk factors establishment.

In conclusion, MG patients hospitalized for COVID-19 may have a more severe course than other hospitalized patients. The baseline immunosuppressive therapy is not necessarily associated with worse outcomes in these patients; thus, its maintenance is advised. Furthermore, immunotherapy for MG exacerbation seems to be safe and must be considered in this context.

## Data Availability Statement

The raw data supporting the conclusions of this article will be made available by the authors, without undue reservation.

## Author Contributions

AC-F, AS, and EZ contributed to the conception and design of the study. AC-F, AS, EZ, and EE contributed to the drafting of the manuscript and table design. All authors contributed to the acquisition, analysis of data, and critical revision of the manuscript.

## Conflict of Interest

The authors declare that the research was conducted in the absence of any commercial or financial relationships that could be construed as a potential conflict of interest.
